# A propos d’une cause inhabituelle de talalgie

**DOI:** 10.11604/pamj.2017.26.168.11553

**Published:** 2017-03-24

**Authors:** Hicham Bousbaa, Mohammed Ouahidi, Mourad Bennani, Tawfik Cherrad, Hassan Zejjari, El Houssine Kasmaoui, Jamal Louaste, Khalid Rachid, Laarbi Amhajji

**Affiliations:** 1 Department of Orthopaedics & Traumatology, Military Hospital Moulay Ismail, BP 50000 Meknes, Morocco

**Keywords:** Tumeur, lipome, calcanéum, douleur plantaire, Tumor, lipoma, calcaneus, plantar pain

## Abstract

Une talalgie est l'une des symptômes les plus courants du pied. LeLipome intra-osseuse est l'un des plus rares tumeurs osseuses trouvées dans le calcanéum. Nous rapportons une observation d’un patient s’est présenté à la consultation avec des douleurs intermittentes chroniques et spontanées du talon, chez qui les investigations cliniques et radiologiques ont retenu un diagnostic d’un lipome du calcanéum.Le but de cet article est de sensibiliser les cliniciens à l'existence de cette lésion inhabituelle.

## Abstract (en)

Talalgie is one of the most common symptoms involving the foot. Intra-bone lipoma is one of the most rare bone tumors of the calcaneus. We report the case of a patient presenting with intermittent and spontaneous chronic heel pain. Clinical and radiological examinations confirmed the diagnosis of lipoma of the calcaneus. This study aimed to educate clinicians about the existence of this rare lesion.

## Introduction

Une talalgie est l´une des symptômes les plus courants du pied [1]. Le plus souvent causée par unefasciiteplantaire ; une fracture de stress; unetendinite; une arthrite, soit une irritation neurologique, mais rarementen rapport avec des lésions lytiques bénignes du calcanéum, qui sont généralement asymptomatiques [2]. Le Lipome intra-osseuse est l´un des plus rares tumeurs osseuses trouvées dans le calcanéum [3, 4]. Nous rapportons une observation d’un patient s’est présenté à la consultation avec des douleurs intermittentes chroniques, et spontanées du talon, chez qui les investigations cliniques et radiologiques ont retenu un diagnostic d’un lipome du calcanéum. Le but de cet article est de sensibiliser les cliniciens à l´existence de cette lésion inhabituelle.

## Patient et observation

Un Homme âgé de 40 ans, sans aucun antécédent traumatique de la cheville, s’est présenté à la consultation avec des douleurs intermittentes du talon gauche depuis 1 mois. La douleur augmente après l´activité sportive et après la station debout prolongée. Cette douleur disparait au repos et elle ne réveillé pas le patient la nuit. L´examen physique était normal ainsi que le bilan biologique.

**Figure 1:**
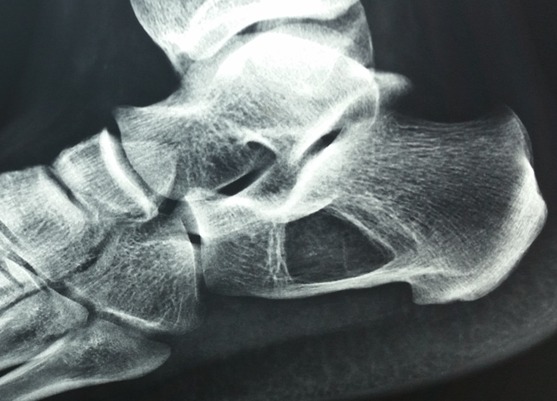
Radiographie de profil de la cheville montrant la lacune calcanéenne

**Figure 2:**
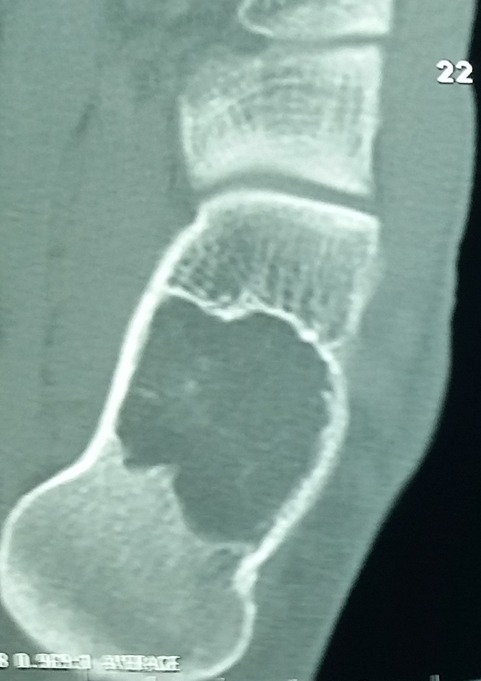
La tomodensitométrie (TDM)de la cheville montrant une lésion lytique bien circonscrite, avec des calcifications centrales

La Radiographie de profil de la cheville gauche a montré une lésion lytique nettement définie siégeant dans le calcanéum, avec des frontières sclérosées, située à la base du calcanéum (le triangle de Ward). Aucune violation corticale ou réaction périostéen’a été noté (Figure 1). La radiographie a suggér un processus bénin, et de nombreux diagnostics ont été évoqués, y compris l´infarctus osseux, la dysplasie fibreuse, l’enchondrome, l’ostéoblastome, etenfin un simple kyste osseux. La tomodensitométrie (TDM) a été réalisée en complément (Figure 2) et a révélé une lésion lytique bien circonscrite, avec des calcifications centrales. L´expansion médullaire légère a été notée sans aucune violation corticale; suggérant le diagnostic d´une calcanéenne lipome intra-osseuse.

Le patient a été mis sous traitement symptomatique avec décharge du talon, etune surveillance régulière en consultation; avec une bonne évolution clinque: à 3 mois il a pu reprendre toutes ces activités notamment sportives.

## Discussion

Le lipome intra-osseuse est une tumeur bénigne rare [1–3], représentant moins de 0,1% des tumeurs osseuses primaires [4]. Elle affecte les os longs, le plus souvent dans les extrémités inférieures, en particulier métaphyse fémorale, tibia, péroné et les corps vertébraux, le crâne, mais aussi la mâchoire et les côtes qui peuvent également être affectés [2]. L´emplacement au calcanéum est estimé à 8% [2], le plus souvent à la base du col du calcanéum (le triangle de Ward) [3]. Les hommes sont touchés dans deux cas sur trois, avec une prédilection pour les adultes entre 30 et 60 ans [3]. Les Lipomes intra-osseux sont généralement asymptomatiques, de découverte fortuite lors d´un examen radiologique dans 40% des cas [4]. Dans les autres cas, la douleur est le symptôme le plus commun et est lié à la position debout ou de l´exercice [5].

L´étiologie de lipome intra-osseuse est inconnue ; de nombreuses théories ont été envisagées : une réaction osseuse secondaire post-traumatique; l’infarctus osseux de la graisse avec métaplasie; l’infection et l’étiologie tumorale car c’est une tumeur bénigne [5]. Enfin, à l´heure actuelle la plupart des auteurs pensent que lipome intra-osseuse est une tumeur primaire de la graisse de moelle osseuse [6–11].

Radiographie simple est très évocatrice du diagnostic montrant une lésion lytique bien circonscrite, avec un nid central de calcification et entourant la jante sclérosée mince. Les lésions peuvent être expansives, sans destruction corticale ou réaction périostée; Toutefois, de rares cas d´effraction corticale ont été rapportés [12]. Les résultats de l´imagerie en coupe sont très suggestifs montrant la nature grasse de la lésion, et permettent d´éviter une biopsie. Ils sont également utiles dans la détection des fractures pathologiques et l´exclusion d´une masse de tissu mou correspondante [13] .

Au scanner, un lipome intra-osseuse est considérée comme une lésion lytique bien définie, avec le équivalentes à celles de la graisse, et l´hétérogénéité de l´unité Hounsfield négative résultant de la nécrose adipeuse, une hémorragie et la calcification dystrophique [14]. L´IRM est une excellente méthode pour démontrer complications comme la nécrose adipeuse, ou un hématome intra-lésionnelle [15].

Le diagnostic différentiel doit inclure fibreuse infarctus osseux, la dysplasie fibreuse, enchondrome, ostéoblastome, simple kyste osseux, et d´autres lésions comme chondrosarcome, liposarcome [14, 15]. Sur le plan histologique, il est un tissu adipeux mature avec calcifications intralésionnelles [16]. Les cas symptomatiques peuvent être traités par curetage et greffe osseuse [16]. Les lésions non ou peudouloureusespeuvent être traitées de façon conservatrice [17], parce que le but de la chirurgie est de soulager la douleur et d´empêcher une fracture pathologique. Aucun cas de récurrence n’a été rapporté dans la littérature [6, 16, 17].

## Conclusion

Les talalgies sont très fréquentes en pratique quotidienne. Le kyste osseux intra calcanéen en est une cause rare. Le traitement peut être conservateur ou chirurgicale après une certitude diagnostic par la TDM ou mieux l’IRM.
